# Prevalence of and Risk Factors for Oral Human Papillomavirus Infection among HIV-Positive and HIV-Negative People Who Inject Drugs

**DOI:** 10.1371/journal.pone.0143698

**Published:** 2015-11-24

**Authors:** Hilary A. Robbins, Christina E. Fennell, Maura Gillison, Weihong Xiao, Yingshi Guo, Alicia Wentz, Gregory D. Kirk, Shruti H. Mehta, Gypsyamber D’Souza

**Affiliations:** 1 Department of Epidemiology, Johns Hopkins Bloomberg School of Public Health, Baltimore, Maryland, United States of America; 2 Spelman College, Atlanta, Georgia, United States of America; 3 Ohio State University Comprehensive Cancer Center, Columbus, Ohio, United States of America; British Columbia Centre for Excellence in HIV/AIDS, CANADA

## Abstract

**Background:**

Human papillomavirus (HPV) causes most oropharyngeal cancers in the United States. Oral HPV prevalence is associated with immunosuppression, and drug use can be immunosuppressive, but the epidemiology of oral HPV among people who use drugs is not well described.

**Methods:**

We enrolled men and women with a current or prior history of injection drug use in this cross-sectional sub-study within the AIDS Linked to the Intravenous Experience (ALIVE) cohort. We tested oral rinse samples for 37 types of HPV DNA and collected self-reported risk factor information. We compared oral HPV prevalence across categories using chi-squared statistics and multivariable logistic regression.

**Results:**

Among 199 subjects, 32% were HIV-positive (median CD4 count 384 cells/μL), 90% were Black, 56% had less than a high school education, 17% had recently used injection drugs, and the median age was 54 years. Most had performed oral sex (82%) but had fewer than 5 lifetime partners (58%). The prevalence of any oral HPV was 29%, and of any oncogenic oral HPV was 13%. Oral HPV prevalence was high among both heterosexual men (30%) and women (20%). After adjustment, odds of oral HPV were increased among HIV-positive individuals with a low CD4 count (<350 cells/μl, aOR = 2.7, 95%CI = 1.2–6.4, vs. HIV-negative individuals), but not among HIV-positive individuals with a higher CD4 cell count. Odds were also elevated for those who had recently performed oral sex on a woman (aOR = 2.2, 95%CI = 1.01–4.6) and, even after this adjustment, among bisexual/lesbian females (aOR = 5.6, 95%CI = 1.4–23, vs. heterosexual females). Oral HPV prevalence was not associated with vaginal sex, performing oral sex on a man, or recent drug use.

**Conclusions:**

Recent drug use was not associated with oral HPV prevalence in our study. However, despite modest numbers of sexual partners, the prevalence of oral HPV among this largely Black population with lower socioeconomic status was high.

## Introduction

There are more than 45,000 new cases of oropharyngeal squamous cell carcinoma (OPC) each year in the United States [1] and more than half are caused by human papillomavirus (HPV) [2]. Patients with HPV-related OPC are more likely to be Caucasian and of high socioeconomic status than other head and neck cancer patients [3]. Risk factors for oral HPV are not well understood, and most studies have focused on select populations including men [4,5], men who have sex with men [6,7], HIV-positive individuals [7,8], young adults [9–12], and primarily Caucasian subjects [7–10].

The prevalence of oral HPV is approximately 7% in the general U.S. population [13], with most infections clearing within 1 year [4,14]. Recent research suggests that HIV-related immunosuppression may increase risk of oral HPV acquisition [14]. Use of illicit drugs, including opiates, cocaine, nicotine, marijuana, and alcohol, can have immunosuppressive effects that may lead to increased risk of some infections [15]. However, it is unclear whether illicit drug use affects oral HPV infection. In this study, we sought to evaluate the prevalence of and risk factors for oral HPV infection among a cohort of primarily heterosexual, Black, HIV-positive and HIV-negative people who inject drugs in Baltimore, Maryland.

## Methods

Participants for this cross-sectional analysis were enrolled as a convenience sample of subjects from the AIDS Linked to the Intravenous Experience (ALIVE) cohort study in Baltimore, Maryland, which includes men and women with a current or prior history of injection drug use [16]. All ALIVE participants who were seen in the study clinic in February or March 2015 were eligible to enroll in this sub-study, and 199 out of 200 individuals approached agreed to participate. Each participant answered an interviewer-administered risk factor survey for this sub-study, in addition to providing risk factor information routinely collected during ALIVE study visits, and also provided a 30-second oral rinse and gargle sample using Scope® mouthwash. Oral DNA was isolated by use of a magnetic bead-based automated platform (QIAsymphony SP, Qiagen) as previously described [17]. Each sample was tested for 37 types of HPV DNA using PCR with PGMY09/11 primer pools followed by type specification with reverse line blot hybridization using the Roche linear array. HPV types 16, 18, 31, 33, 35, 39, 45, 51, 52, 56, 58, 59, 68, and 73 were considered oncogenic [18–20], and non-oncogenic types included HPV 6, 11, 26, 40, 42, 53–55, 61, 62, 64, 66, 67, 69–72, 81–84, 89 (CP6108), and IS39. This study was approved by the Johns Hopkins Bloomberg School of Public Health Institutional Review Board (H.34.01.11.19.A1 and H.34.99.05.04.A1), and all participants provided written informed consent.

In univariate analyses, we explored differences in the prevalence of oral HPV (i.e., presence of any of the 37 types for which we tested) across demographic and risk factor categories. We tested for statistically significant differences across groups using chi-square statistics. For variables that were collinear, we selected only one variable to include in the final model based on univariate results and prior literature. This included selecting current CD4 cell count instead of nadir CD4 cell count, and number of recent oral sex partners instead of recent open-mouth kissing partners. The final multivariable logistic model included age, race/ethnicity, HIV/CD4 cell count, sex and sexual orientation, and the number of women on whom the participant had recently performed oral sex.

## Results

Our study included 199 people with a past and/or current history of injection drug use, including 64 HIV-positive and 135 HIV-negative participants (Table 1). There were both heterosexual males (66%) and heterosexual females (22%), as well as some bisexual/gay males (6%) and bisexual/lesbian females (6%). Most participants were non-Hispanic Black (90%), aged 50–59 years (59%), and had less than a high school education (56%). Among HIV-positive participants, nearly half had a current CD4 cell count of less than 350 cells/μL (Table 1). Although all participants had a history of injection drug use, only 17% of participants were currently using injection drugs, while 82% and 46% were currently using cigarettes and alcohol, respectively.

**Table 1:** Characteristics of participants and prevalence of oral HPV among 199 people who inject drugs in Baltimore City.

Characteristic	Number of participants (%)	Oral HPV prevalence	p-value
Overall	199 (100)	29%	
Age, years			0.55
<50	45 (23)	33%	
50–59	118 (59)	29%	
≥60	36 (18)	22%	
Race/ethnicity			0.07
Non-Hispanic Black	180 (90)	31%	
White/ other race	19 (10)	11%	
Education			0.41
Less than high school	111 (56)	31%	
High school or higher	87 (44)	25%	
Sex and sexual orientation			0.06
Heterosexual female	44 (22)	20%	
Heterosexual male	132 (66)	30%	
Bisexual/lesbian female	12 (6)	58%	
Bisexual/gay male	11 (6)	18%	
HIV and current CD4 cell count			0.04
HIV-negative	135 (68)	26%	
HIV-positive, CD4 ≥ 350	35 (18)	23%	
HIV-positive, CD4 < 350	29 (15)	48%	
HIV and nadir CD4 cell count			0.02
HIV-negative	135 (68)	26%	
HIV-positive, nadir CD4 ≥ 200	25 (13)	16%	
HIV-positive, nadir CD4 < 200	39 (20)	46%	
Cigarette use in last 6 months			0.42
No	35 (18)	34%	
Yes	164 (82)	27%	
Cigarettes per day in last 6 months			0.69
None	35 (18)	34%	
1–9	81 (41)	28%	
≥10	83 (42)	27%	
Alcohol use in last 6 months			0.60
No	107 (54)	27%	
Yes	92 (46)	30%	
Marijuana use in last 6 months			0.76
No	170 (85)	28%	
Yes	29 (15)	31%	
Any injection drug use^ in last 6 months			0.61
No	164 (83)	28%	
Yes	34 (17)	32%	
Any crack/cocaine* use in last 6 months			0.57
No	152 (76)	28%	
Yes	47 (24)	32%	
Any heroin* use in last 6 months			0.78
No	171 (87)	28%	
Yes	26 (13)	31%	
Lifetime number of vaginal sex partners			0.64
0–5	52 (26)	23%	
6–15	57 (29)	30%	
16–50	56 (28)	34%	
>50	34 (17)	26%	
Ever performed oral sex			0.10
No	35 (18)	17%	
Yes	164 (82)	31%	
*Lifetime* number of people (male or female) performed oral sex on			0.12
None	35 (18)	17%	
1–4	79 (40)	27%	
≥5	85 (43)	35%	
*Lifetime* number of *men* performed oral sex on			0.74
None	148 (74)	28%	
1–4	28 (14)	25%	
≥5	23 (12)	35%	
*Lifetime* number of *women* performed oral sex on			0.22
None	73 (37)	22%	
1–4	64 (32)	30%	
≥5	62 (32)	35%	
Performed oral sex on a *man* in last 6 months			0.99
None	178 (89)	29%	
1 or more	21 (11)	29%	
Performed oral sex on a *woman* in last 6 months			0.04
None	149 (75)	25%	
1 or more	50 (25)	40%	
Open-mouth kissed anyone in last 6 months			0.01
None	115 (58)	22%	
1 or more	83 (42)	39%	

^ Any injection drug use includes injected cocaine, heroin, speedball, crystal meth, and painkillers.

* Any crack/cocaine use includes cocaine that was injected, smoked (i.e., crack), or snorted. Any heroin use includes heroin that was injected, smoked, or snorted.

The overall prevalence of any oral HPV was 29% (57/199), and prevalence of any oncogenic oral HPV was 13% (26/199). The most common oral HPV types detected were HPV55 (4.5%), HPV45 (4.0%), and HPV59 (3.0%), with HPV16 and HPV18 detected among 2.5% and 0.50% of participants, respectively. None of the types of recent drug use examined was associated with oral HPV prevalence. This included null associations for any recent use of: marijuana (OR = 1.1, 95% CI 0.49–2.7); cocaine whether smoked/snorted (OR = 0.83, 95% CI 0.37–1.8) or injected (OR = 1.6, 95% CI 0.54–4.6); heroin whether smoked/snorted (OR = 1.2, 95% CI 0.46–3.1) or injected (OR = 1.1, 95% CI 0.43–2.9); or recent injection of any drug (OR = 1.2, 95% CI 0.55–2.7).

Risk factors for oral HPV prevalence in univariate analyses included immunosuppression, sexual behavior, sexual orientation, and race/ethnicity (Table 1). HIV-positive participants with a low CD4 count (<350 cells/μL) had higher oral HPV prevalence (48%) than HIV-negative (26%) or less immunosuppressed HIV-positive participants (23%, p = 0.04). A similar pattern was present for nadir CD4 count (Table 1). Oral HPV prevalence was associated with recent but not lifetime sexual behavior. Specifically, recently performing oral sex on a *woman* was associated with higher oral HPV prevalence (40% vs. 25%, p = 0.04), but recently performing oral sex on a *man* was not (29% vs. 29%, p = 0.99). Other risk factors included higher prevalence among bisexual/lesbian females (58%) than other groups (p = 0.06) and among non-Hispanic Blacks than those of any other race/ethnicity (31% vs. 11%, p = 0.07). Oral HPV was not associated with any of the types of drug use that we examined, namely, tobacco, alcohol, marijuana, crack/cocaine, heroin, and any injection drug use (all p>0.4).

Multivariable risk factors for prevalent oral HPV are described in Table 2. Odds of oral HPV remained significantly increased among HIV-positive individuals with a CD4 count <350 cells/μL (aOR = 2.7, 95% CI 1.2–6.4, vs. HIV-negative individuals) and those who had performed oral sex on a woman in the prior 6 months (aOR = 2.2, 95% CI 1.01–4.6). After adjusting for other risk factors including this sexual behavior, bisexual/lesbian females remained at increased odds of oral HPV (aOR = 5.6, 95% CI 1.4–23) compared to heterosexual females, but males did not (aOR = 1.4, 95% CI 0.55–3.4). Odds of oral HPV remained significantly increased among Blacks compared to non-Blacks in our study (aOR = 5.1, 95% CI 1.04–25).

**Table 2:** Multivariable logistic model for oral HPV among 199 people who inject drugs in Baltimore City.

Characteristic	Odds ratio (95% CI)	p-value
Age, years		0.276
<50	Reference	
50–59	0.62 (0.27–1.4)	
≥60	0.41 (0.14–1.2)	
Race/ethnicity		0.045
White / other race	Reference	
Non-Hispanic Black	5.1 (1.04–25)	
HIV and current CD4 cell count		0.049
HIV-negative	Reference	
HIV-positive, CD4 ≥ 350	0.82 (0.32–2.1)	
HIV-positive, CD4 < 350	2.7 (1.2–6.4)	
Performed oral sex on a woman in last 6 months		0.047
No	Reference	
Yes	2.2 (1.01–4.6)	
Sex and sexual orientation		0.052
Heterosexual female	Reference	
Bisexual/lesbian female	5.6 (1.4–23)	
Male, any sexual orientation	1.4 (0.55–3.4)	

## Discussion

In this cross-sectional study of 199 people who inject drugs, recent drug use was not associated with oral HPV prevalence. Despite modest numbers of sexual partners, the prevalence of oral HPV among this population of minority participants with lower socioeconomic status was high. Risk factors for oral HPV infection included HIV-related immunosuppression and recently performing oral sex on a woman.

The overall prevalence of oral HPV (29%) found in our study is much higher than in the general population (7%) [13]. This was influenced by high oral HPV prevalence among HIV-positive participants with a low CD4 count (15% of our study population, with an oral HPV prevalence of 48%). However, even among HIV-negative individuals in our study, oral HPV prevalence (23%) was higher than in the general population. Our study population consisted largely of heterosexual men, with most aged 50–59 years, and these groups have the highest incidence of OPC [21] and the highest prevalence of oral HPV [13,22,23]. Participants in our study were also largely Black, a group in which oral HPV prevalence has not been well explored, although some previous studies have suggested higher oral HPV prevalence of Blacks than Caucasians in unadjusted analyses [13]. In our study, the prevalence of oral HPV remained elevated among Blacks even after adjustment for a number of sexual behaviors. However, residual confounding is possible, and a previous larger study by our group suggested that racial disparities in oral HPV prevalence are explained by differences in sexual behavior [24].

We found no evidence that illicit drug use itself is associated with oral HPV, consistent with another small study that considered the effects of marijuana, heroin, cocaine, crack, and speedball use on oral HPV [25]. This differs from findings for cervical HPV infection, which has been reported to be elevated among women who use crack or marijuana [26]. We also did not observe an association between oral HPV prevalence and tobacco use, which has been reported in several larger previous studies [5,6,27].

The association of HIV-related immunosuppression (CD4<350 cells/μL) with oral HPV prevalence observed in this study is consistent with several other studies [7,8,14,28]. We found no evidence for higher prevalence of oral HPV among participants with well-controlled HIV (CD4≥350), which suggests that effective HIV treatment may help to improve oral HPV clearance or reduce HPV incidence, as previously reported [14].

Our findings support the hypothesis that performing oral sex on a woman confers higher risk of acquiring oral HPV than performing oral sex on a man [23,27,29]. It is interesting that risk among bisexual/lesbian women remained elevated even after adjusting for number of recent female oral sex partners, though this finding should be considered preliminary as there were a limited number of lesbian/bisexual women in this study. Elevated oral HPV prevalence among bisexual/lesbian females could be due to differences in their number of lifetime female oral sex partners, age at sexual initiation, or other differences in sexual behavior that might influence their exposure to or development of antibodies to HPV. Adolescent and young adult lesbians have been less likely to initiate HPV vaccination than young heterosexual females in some studies [30,31], and the high oral HPV prevalence found in our study supports the importance of HPV vaccination in this population.

Strengths of our study include a high participation rate, detailed risk factor information, and centralized laboratory testing of all samples. We were limited by our sample size, which may have prevented us from detecting small associations between oral HPV and drug use, if they exist. Our study population was also fairly homogenous with respect to race/ethnicity and age, so we were unable to adequately describe variation in oral HPV prevalence across these characteristics. Though we interpreted the detection of HPV DNA to indicate HPV infection, it is also possible that in some cases we detected HPV DNA in saliva samples that did not actually represent infection. Finally, because our study is cross-sectional, we have described patterns in prevalence of oral HPV but we cannot address HPV incidence or clearance.

In conclusion, our results suggest that HIV-related immunosuppression and performing oral sex on a woman are strong risk factors for oral HPV, while drug use was not associated with oral HPV. Nevertheless, we observed high oral HPV prevalence among people who currently or formerly used injection drugs, implying that this population may have increased risk of HPV-related OPC. Additional research will be needed to determine how many of these infections persist long-term and may represent cancer risk, and whether this population could benefit from increased emphasis on HPV vaccination or perhaps oral cancer screening.

## Supporting Information

S1 File Data file with information on variables from the final multivariate model in Table 2. (CSV)
